# MEMS Gyroscope Temperature Compensation Based on Drive Mode Vibration Characteristic Control

**DOI:** 10.3390/mi10040248

**Published:** 2019-04-14

**Authors:** Min Cui, Yong Huang, Wei Wang, Huiliang Cao

**Affiliations:** 1 Science and Technology on Electronic Test & Measurement Laboratory, North University of China, Tai Yuan 030051, China; cmcm_1980930@163.com (M.C.); huangyong9608@163.com (Y.H.); wangweinuc@163.com (W.W.); 2 School of Instrument and Electronics, North University of China, Tai Yuan 030051, China

**Keywords:** dual-mass MEMS gyroscope, temperature compensation, scale factor, bias, drive mode, amplitude controlling

## Abstract

In this paper, a novel temperature compensation method for a dual-mass MEMS gyroscope is proposed based on drive mode vibration characteristic compensation using a temperature variable resistor. Firstly, the drive and sense modes of the gyroscope re analyzed and investigated, and it is found that the scale factor is proportional to the drive mode amplitude controlling reference voltage. Then, the scale factor temperature compensation method is proposed, and a temperature variable resistor is utilized to compensate the drive amplitude working point and make it change with temperature. In addition, the temperature compensation circuit is designed and simulated. After that, the temperature bias drift is compensated in a modular output. The experimental results show that scale factor and bias variation during the temperature range from −40 °C to 60 °C decrease from 3.680% to 1.577% and 3.880% to 1.913%, respectively. In addition, the bias value improves from 103.395 °/s to 22.478 °/s (optimized 78.26%). The bias stability and angular rate walking parameter are also optimized to 45.97% and 16.08%, respectively, which verify the method proposed in this paper.

## 1. Introduction

An MEMS gyroscope detects input angular rate signals by using the Coriolis force [1,2]. The precision of a MEMS gyroscope has improved a great deal over the past years, but poor temperature characteristics limit its application. This is due to three aspects: the silicon structure has a high temperature coefficient; the silicon-glass bonding style and package cause thermal residual stress; and the monitoring system sometimes drifts with temperature. So, it can be concluded that the methods of MEMS gyroscope temperature compensation should comprise four aspects, as follows. 

Structure improvement: A temperature compensation fork was arranged in the MEMS gyroscope structure to compensate the influence of temperature in [3], and in the temperature range of −20 to 80 °C, the maximum relative error of the resonant frequency was reduced from 16.3% to 3.1%. Reference [4] described the MEMS gyroscope structure compensation with a disturbance estimator and indicated that the architecture’s imperfect fabrication and asymmetry can also decrease the temperature characteristics. A thermal stress structure was utilized in a cylindrical gyroscope to improve temperature performance in [5], and the open-loop temperature drift rate was reduced by about 2 thirds after the structure was optimized. Two epoxy materials were filled between the structure substrate and package to decrease the quality factor temperature coefficient of the gyroscope in [6], and the temperature coefficient of the drive and sense mode resonant frequencies were 124.1 ppm/°C and −106.9 ppm/°C. In [7], a novel stress compensation method was proposed to assist conventional temperature compensation to improve the long-term drift of MEMS gyroscopes, and a long-term (around 3 hours) test showed that the angle random walk was 0.15°/√h, and bias instability was 1°/h. Reference [8] presented an architecture which had a higher temperature stability and robustness; in this structure, the drive-mode operational frequency and the sense-mode bandwidth can be set independently, and the uncompensated temperature coefficients of bias and scale factor were 313 °/(h·°C) and 351 ppm/°C, respectively. Reference [9] presented an example of temperature compensation in the silicon resonator’s architecture by using an I-shaped beam. The structure improvement requires design, fabrication, packaging and other processes, which requires a long-term research cycle.

Software temperature compensation: Multi-resolution analysis was employed to compensate temperature drift and de-noising. In [10], the radial basis function (RBF) neural network method was used in an ASDXRS150 sensor with the −40 °C to 60 °C temperature range, and the compensation results showed that the method improved the maximum gyro error and mean square error value by 17.6% and 31.2%, respectively. A wavelet transform algorithm was reported in [11] to reduce the temperature drift of an MEMS gyroscope. Reference [12] employed lifting wavelet transform method to improve the noise performance of an MEMS gyroscope. Reference [13] processed the output data with a linear compensation algorithm by using the relationship between the temperature inside the gyroscope’s shell and the output data; after the compensation, the temperature coefficients of bias stability improved from 229.1 °/(h·°C) to 35.7 °/(h·°C). Reference [14] introduced an integrated electromechanical–thermal error model and employed a least-squares algorithm to compensate the bias drift caused by temperature and acceleration. Variational mode decomposition (VMD) and genetic-Elman neural network methods are used to compensate temperature drift and denoising in [15] and [16]. A radial basis function neural network based on the genetic algorithm with Kalman filter was reported in [17], and the bias stability and angle random walk of the MEMS gyroscope was improved from 178 °/h to 1.6 °/h and 5.89°/√h to 0.71°/√h, respectively, within the −40 °C to 60 °C temperature range. Reference [18] used a high-order polynomial to compensate the bias of a double H quartz tuning fork gyroscope on a digital signal processing platform; the variation of the bias in the range −40 °C to 80 °C reduced from 300 mV to 0.2 mV because of the compensation. The software compensation methods usually cannot deal with the output data online and lack real-time capability; thus, they are better used in the data analysis research area.

Hardware temperature compensation: A hardware temperature compensation method based on a circuit amplifier was proposed in [19], and through the temperature variable resistor temperature compensation, the scale factor and temperature bias coefficient were optimized from 693 ppm/°C to 250 ppm/°C and from 103.89 °/(h·°C) to 9.70 °/(h·°C), respectively. Bandwidth temperature compensation methods were proposed in [20] and [21], and the bandwidth was improved from 13 Hz to more than 100 Hz. The hardware temperature compensation methods have better real-time characteristics and a short development cycle. 

Temperature-control: Reference [22] proposed a temperature-control system to steady the ambient temperature to improve the gyroscope’s temperature performance. On-chip temperature control technology was utilized to decrease the MEMS gyroscope’s temperature drift in [23]; the micro thermal resister, heater and a thermal isolate package were employed to form the on-chip temperature control system. Those methods require a good deal of power consumption and are not fit for low-power application regions.

In this paper, a temperature compensation method based on drive mode vibration characteristics is proposed to improve gyro precision, and the results are analysed. This paper is organized as follows: the structure of the MEMS gyroscope and the monitoring system are introduced in Section 2; the temperature compensation method is shown in Section 3; Section 4 shows the temperature experiment; and finally, the conclusion is given in Section 5.

## 2. Dual-Mass MEMS Gyroscope

### 2.1. Dual-Mass MEMS Gyroscope Structure

**Figure 1:**
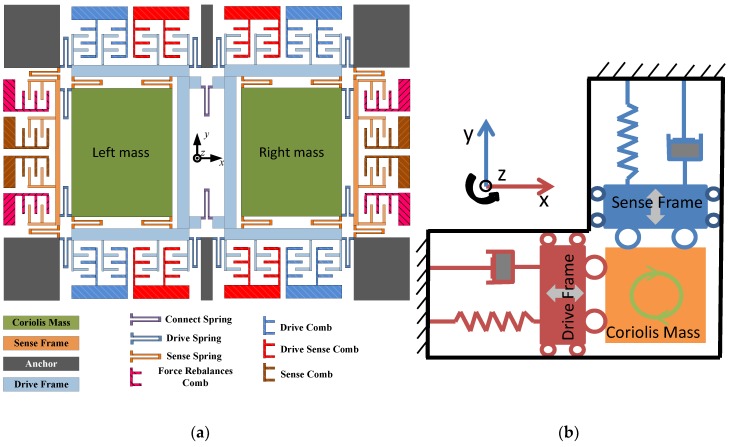
Dual-mass MEMS gyroscope structure (**a**) and its mechanical model (**b**).

The dual-mass and turning-fork structure of the MEMS gyroscope is investigated in this paper. The structure of the turning-fork gyroscope is shown in Figure 1. The structure is formed by two modes: the drive mode (including drive frame, drive springs and drive comb) and sense mode (including sense frame, sense springs and sense comb). The Coriolis mass is the public part of these two modes, and it has two degrees of freedom (along the *x* axis and *y* axis). This structure is fully decoupled, which is better for decreasing the quadrature error [24]; drive and sense modes are not coupled with each other, and only the mass is the public part. The structure has two masses, and they vibrate inversely along the *x* axis when the drive mode (only can move along *x* axis) is stimulated by electrostatic force. Following from the angular rate input *Ω_z_* around the *z* axis, the vibrating mass generates a Coriolis force and transfers it into the sense frame movement (in *y* axis), which can be detected by the monitor circuit.

**Figure 2:**
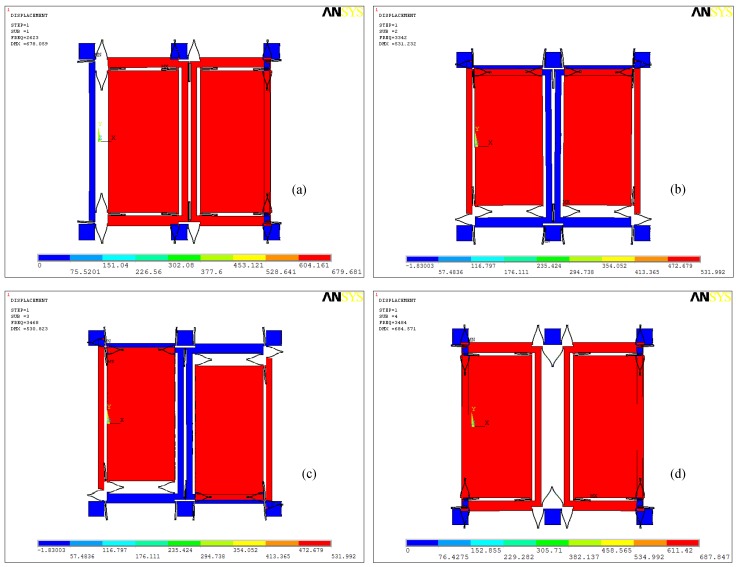
The first four orders modes of the dual-mass sensing mode coupling MEMS gyroscope structure: (**a**) Drive in-phase mode (first mode) with frequency *ω_x_*_1_; (**b**) sensing in-phase mode (second mode) with frequency *ω_y_*_1_; (**c**) sensing anti-phase mode (third mode) with frequency *ω_y_*_2_; (**d**) Drive anti-phase mode (fourth mode) with frequency *ω_x_*_2_.

The first four order modes of the structure are analyzed and shown in Figure 2. Figure 2a shows the 1st mode (the simulation resonant frequency is 2623 Hz), the drive in-phase mode, and that the left and right masses move in the same direction along *x* axis; Figure 2b shows the 2nd mode (the simulation resonant frequency is 3342 Hz), the sensing in-phase mode, and that the left and right masses move in same direction along *y* axis; Figure 2c shows the 3rd mode (the simulation resonant frequency is 3468 Hz), sensing anti-phase mode, and the left and right masses moving in an inverse direction along the *y* axis; finally, Figure 2d shows the 4th mode (the simulation resonant frequency is 3484 Hz), drive anti-phase mode, and left and right masses moving in an inverse direction along the *x* axis. 

The drive anti-phase is expected to be the drive mode. So, left and right Coriolis masses both have two degrees of freedom (along the *x* and *y* axes). The drive frame has one degree of freedom (along the *x* axis), and the sense frame has one degree of freedom (along the *y* axis). The resonant frequency and *Q* value of one manufactured structure are tested under different temperatures, as shown in Table 1.

### 2.2. Dual-Mass MEMS Gyroscope Structure Working Principle

The dynamic equation of the MEMS gyroscope structure resonator can be given as follows [21]:

(1) $$\left[\begin{array}{ccc} m_{x} & 0 & 0\\ 0 & m_{y} & 0\\ 0 & 0 & m_{y} \end{array}\right]\left[\begin{array}{l} \ddot{x}\\ {\ddot{y}}_{1}\\ {\ddot{y}}_{2} \end{array}\right]+\left[\begin{array}{ccc} \frac{\omega_{x2}m_{x}}{Q_{x2}} & 0 & 0\\ 0 & \frac{\omega_{y1}m_{y}}{Q_{y1}} & 0\\ 0 & 0 & \frac{\omega_{y2}m_{y}}{Q_{y2}} \end{array}\right]\left[\begin{array}{l} \dot{x}\\ {\dot{y}}_{1}\\ {\dot{y}}_{2} \end{array}\right]+\left[\begin{array}{ccc} \omega_{x2}^{2}m_{x} & 0 & 0\\ 0 & \omega_{y1}^{2}m_{y} & 0\\ 0 & 0 & \omega_{y2}^{2}m_{y} \end{array}\right]\left[\begin{array}{l} x\\ y_{1}\\ y_{2} \end{array}\right]=\left[\begin{array}{l} F_{dx}\sin\left(\omega_{d}t\right)\\ -2m_{c}\Omega_{z}\dot{x}\\ -2m_{c}\Omega_{z}\dot{x} \end{array}\right]$$

where *m_x_*, *m_y_* and *m_c_* are the equivalent masses of the drive mode, sense mode and Coriolis; *x*, *y*_1_ and *y*_2_ are the displacement of the drive mode, sense in-phase mode and sense anti-phase mode, respectively; *Q_x_*_2_, *Q_y_*_1_ and *Q_y_*_2_ are the quality factors of the drive mode, sense in-phase mode and sense anti-phase mode, respectively; *Ω_z_* is the angular rate input; *m_y_* ≈ *m_c_*; and *F_dx_* is the drive mode stimulating magnitude; *ω_d_* is the drive mode stimulating frequency. From the total sense mode displacement, *y* = *y*_1_ + *y*_2_, we obtain the following [20,21]:

(2) $$\left\{ \begin{array}{l} x(t)=\frac{F_{dx}Q_{x2}}{m_{x}\omega_{d}^{2}}\cos(\omega_{d}t)=A_{x}\cos(\omega_{d}t)\\ y_{1,2}(t)=\frac{-2\Omega_{z}F_{dx}Q_{x2}\sin(\omega_{d}t)}{m_{x}\omega_{d}\sqrt{{\left(\omega_{y1,2}^{2}-\omega_{d}^{2}\right)}^{2}+\omega_{y1,2}^{2}\omega_{d}^{2}/Q_{y1,2}^{2}}}=A_{y1,2}\sin(\omega_{d}t) \end{array}\right.$$

Then, the dual-mass MEMS gyroscope structure’s mechanical sensitivity can be expressed as follows:

(3) $$S_{me}=\frac{A_{y1}+A_{y2}}{\Omega_{z}}\approx \frac{-F_{dx}Q_{x2}}{m_{x}\omega_{d}^{2}}\left(\frac{1}{\omega_{y1}-\omega_{x2}}+\frac{1}{\omega_{y2}-\omega_{x2}}\right)=-A_{x}\left(\frac{1}{\Delta \omega_{1}}+\frac{1}{\Delta \omega_{2}}\right)$$

### 2.3. Dual-Mass MEMS Gyroscope Monitoring System

**Figure 3:**
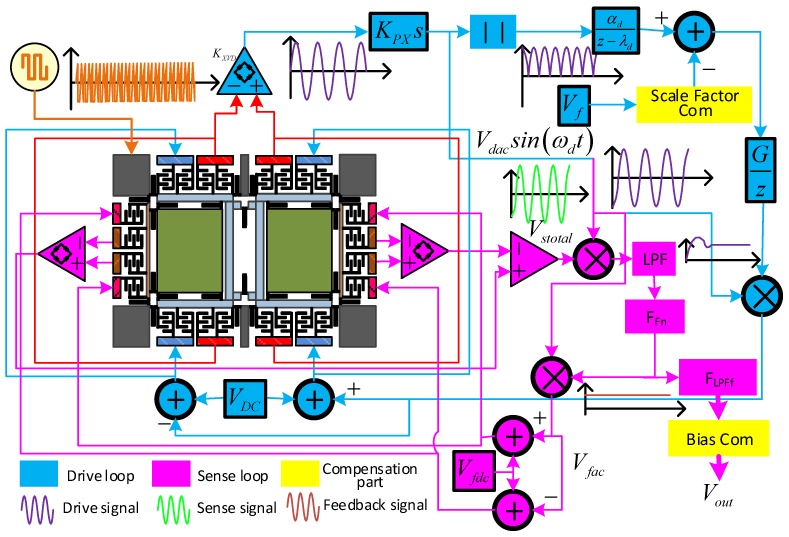
MEMS gyroscope monitoring system schematic.

The dual-mass MEMS gyroscope monitoring system contains a drive closed-loop and sense closed-loop, and the system schematic is shown in Figure 3. In the drive loop (blue color in Figure 3), drive mode displacement *x(t)* is detected by drive sense combs and processed by differential amplifier. Then, the *K_PX_s* module delays the signal phase to 90° to satisfy the phase requirement of the AC drive signal *V_dac_*sin*(ω_d_t)*. After that, the full-wave rectifier and a low-pass filter pick up the amplitude of *V_dac_*sin*(ω_d_t)*. Additionally, *V_dac_* is compared with the reference voltage *V_ref_* by a comparer. Next, a drive closed-loop proportional-integral (PI) controller generates the control signal, which is modulated by *V_dac_*sin*(ω_d_t)*, and then the signal is superposed by *V_DC_* to stimulate the drive mode. 

The sense closed loop (magenta color in Figure 3) employs the same interface as the drive loop circuit. Firstly, the left and right sense frame movement signals are detected separately with differential detection amplifiers, and the output signals are processed by a second differential amplifier to form the sense mode movement signal *V_stotal_*. Then, *V_stotal_* is demodulated by signal *V_dac_*sin*(ω_d_t),* and a demodulated signal *V_dem_* is generated. After that, *V_dem_* passes through the two-order low-pass filter and forms the sense feedback signal, and it is first sent in *F_Fn_* to calculate the control signal. Then, the signal is modulated with *V_dac_*sin*(ω_d_t)* to form the AC feedback signal. Finally, DC voltage *V_fdc_* is superposed with the AC feedback signal to generate the feedback signal. The output level of the low-pass filter *F_LPFf_* is configured to decrease the output noise of the gyroscope.

## 3. Temperature Compensation Method Based on Drive Mode Vibration Characteristic Control

### 3.1. Dual-Mass MEMS Gyroscope Drive Mode Vibration Control System

**Figure 4:**
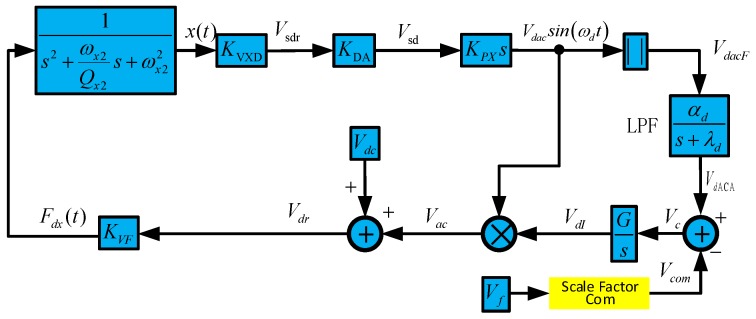
MEMS gyroscope, drive mode closed-loop system.

The dual-mass MEMS gyroscope drive mode closed-loop controlling system employs self-oscillation technology, and the controller is based on automatic gain control (AGC) technology. The control system is shown in Figure 4. The system working state is analyzed as follows [19]:

The drive force *F_dx_*sin*(ω_d_t)* generated by electrostatic force can be expressed as

(4) $$F_{dx}\sin\left(\omega_{d}t\right)=4\frac{\partial C_{d}}{\partial x}V_{dc}V_{ac}$$

where *C_d_* is the capacitance formed by drive combs on one side. By inputting Equations (4) to (1), and expanding *V_ac_*, in according to Figure 4, we obtain

(5) $$\left\{ \begin{array}{l} \ddot{x}+\frac{\omega_{d}}{Q_{x2}}\dot{x}+\omega_{d}^{2}x=4\frac{\partial C_{d}}{\partial x}V_{dc}V_{dI}(t)K_{XVD}K_{DA}K_{PX}\dot{x}\;\\ {\dot{V}}_{dI}(t)=G(V_{com}-V_{dACA})\;\\ {\dot{V}}_{dACA}=\left|K_{XVD}K_{DA}K_{PX}\dot{x}\right|\alpha_{d}-\lambda_{d}V_{dACA}(t)\; \end{array}\right.$$

The drive mode speed and acceleration can be expressed as

(6) $$\left\{ \begin{array}{l} \dot{x}=-A_{x}(t)\omega_{d}\sin(\omega_{d}t)\\ \ddot{x}=-{\dot{A}}_{x}\omega_{d}\sin(\omega_{d}t)-A_{x}(t)\omega_{d}^{2}\cos(\omega_{d}t) \end{array}\right.$$

Substituting Equations (2) and (6) into (5), we then obtain

(7) $${\dot{A}}_{x}=4\frac{\partial C_{d}}{\partial x}V_{dc}V_{dI}(t)K_{XVD}K_{DA}K_{PX}A_{x}(t)-\frac{\omega_{d}A_{x}(t)}{Q_{x2}}$$

Then, applying the average method, considering the average value in one period (*T =* 2π*/ω_d_*) of Equations (5) and (7), we obtain

(8) $$\left\{ \begin{array}{l} {\dot{V}}_{dI}(t)=\frac{1}{T}\int_{0}^{T}G(V_{com}-V_{dACA})dt\;\\ {\dot{\bar{V}}}_{dACA}=\frac{1}{T}\int_{0}^{T}\left|K_{XVD}K_{DA}K_{PX}A_{x}(t)\omega_{d}\sin(\omega_{d}t)\right|\alpha_{d}-\lambda_{d}V_{dACA}(t)dt\;\\ {\dot{\bar{A}}}_{x}=\frac{1}{T}\int_{0}^{T}\left(4\frac{\partial C_{d}}{\partial x}V_{dc}V_{dI}(t)K_{XVD}K_{DA}K_{PX}A_{x}(t)-\frac{\omega_{d}A_{x}(t)}{Q_{x2}}\right)dt\; \end{array}\right.$$

Thus,

(9) $$\left\{ \begin{array}{l} {\dot{\bar{V}}}_{dI}(t)=G(V_{com}-{\bar{V}}_{dACA})\;\\ {\dot{\bar{V}}}_{dACA}=\frac{2}{\pi }{\bar{A}}_{x}\omega_{nx}K_{DA}K_{PX}\alpha_{d}\left|K_{XVD}\right|-\lambda_{d}{\bar{V}}_{dACA}\;\\ {\dot{\bar{A}}}_{x}=4\frac{\partial C_{d}}{\partial x}V_{dc}{\bar{V}}_{dI}(t)K_{XVD}K_{DA}K_{PX}{\bar{A}}_{x}(t)-\frac{\omega_{d}{\bar{A}}_{x}(t)}{Q_{x2}}\; \end{array}\right.$$

Let the right side of Equation (9) be equal to zero; we then obtain

(10) $$\left\{ \begin{array}{l} {\bar{V}}_{dACA0}=V_{com}\\ {\bar{A}}_{x0}=\frac{\pi \lambda_{d}}{2\omega_{d}K_{DA}K_{PX}\alpha_{d}\left|K_{XVD}\right|}\;V_{com}\;\\ {\bar{V}}_{dI0}=-\frac{\partial x\omega_{d}}{4\partial C_{d}Q_{x2}K_{XVD}K_{DA}K_{PX}V_{dc}}\; \end{array}\right.\;$$

Thus, the system has only one stable working condition, and the drive mode amplitude is controlled as per Equation (10), and *V_com_* will be the parameter used to control the drive mode amplitude.

### 3.2. Dual-Mass MEMS Gyroscope Sense Mode Closed-loop System

**Figure 5:**
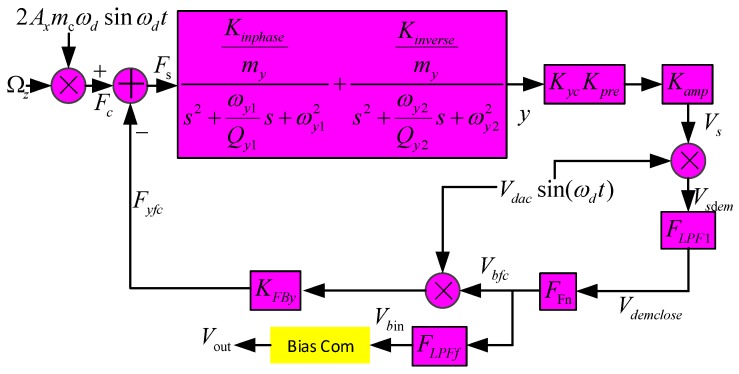
MEMS gyroscope sense mode closed-loop system.

The dual-mass MEMS gyroscope sense closed-loop system schematic is shown in Figure 5.

In Figure 5, it can be seen that the sense real working mode is the superposition of the sense in-phase mode and sense anti-phase mode, which has been already researched in our previous paper [20]. *K_yc_* and *K_pre_* are displaced to the capacitance transform function and pre-amplifier; *K_amp_* is the second differential amplifier; *F_LPF1_* is the second-order low-pass filter; *F_Fn_* is the controller; *K_FBy_* is the voltage–force interface transform coefficient of the force-rebalanced combs; *K_inphase_* and *K_inverse_* are the displaced-voltage transform parameters of the sensing in-phase and anti-phase modes; and *K_inverse_* ≈ 33·*K_inphase_*, *m_c_* ≈ *m_y_*. Thus, we obtain the following equations:

(11) $$\left\{ \begin{array}{l} F_{c}\left(t\right)=2\Omega_{z}\left(t\right)m_{y}A_{x}\omega_{d}\sin\left(\omega_{d}t\right)\\ F_{yfc}\left(t\right)=K_{FBy}V_{bfc}\left(t\right)V_{dac}\sin\left(\omega_{d}t\right)\\ F_{s}\left(t\right)=F_{c}\left(t\right)-F_{yfc}\left(t\right)\\ V_{sdem}\left(t\right)=V_{s}\left(t\right)V_{dac}\sin(\omega_{d}t)\\ V_{bin}=V_{sdem}F_{LPF1}F_{Fn}F_{LPFf} \end{array}\right.$$

After a series of transformations and calculations, the relationship between *V_bin_* and *Ω_z_* (the scale factor expression of the MEMS gyroscope sense closed-loop system) can be expressed as

(12) $$\frac{V_{bin}\left(s\right)}{\Omega_{z}(s)}=\frac{2K_{yc}K_{pre}K_{amp}V_{dac}F_{LPF1}F_{Fn}F_{LPFf}m_{y}A_{x}\omega_{d}G_{sE}}{4m_{y}+K_{yc}K_{pre}K_{amp}V_{dac}^{2}F_{LPF1}F_{Fn}K_{FBy}G_{sE}}$$

where *G_sE_* is the gyroscope sense mode transform equation and can be expressed as

$$G_{sE}=\{\frac{K_{inphase}(s^{2}+\frac{\omega_{y1}}{Q_{y1}}s+\omega_{y1}^{2}-\omega_{d}^{2})}{{\left(s^{2}+\frac{\omega_{y1}}{Q_{y1}}s+\omega_{y1}^{2}-\omega_{d}^{2}\right)}^{2}+{\left(2s\omega_{d}+\frac{\omega_{y1}}{Q_{y1}}\omega_{d}\right)}^{2}}+\frac{K_{inverse}(s^{2}+\frac{\omega_{y2}}{Q_{y2}}s+\omega_{y2}^{2}-\omega_{d}^{2})}{{\left(s^{2}+\frac{\omega_{y2}}{Q_{y2}}s+\omega_{y2}^{2}-\omega_{d}^{2}\right)}^{2}+{\left(2s\omega_{d}+\frac{\omega_{y2}}{Q_{y2}}\omega_{d}\right)}^{2}}\}$$

*F_Fn_* is designed to satisfy the following equation:

$$4m_{y}<<K_{yc}K_{pre}K_{amp}V_{dac}^{2}F_{LPF1}F_{Fn}K_{FBy}G_{sE}$$

Additionally, we obtain the following equation from Equation (12):

(13) $$\frac{V_{bin}\left(s\right)}{\Omega_{z}(s)}=\frac{2F_{LPFf}m_{y}A_{x}\omega_{d}}{V_{dac}K_{FBy}}$$

Then, substituting Equation (10) into (13), the MEMS gyroscope scale factor can be obtained:

(14) $$\frac{V_{bin}\left(s\right)}{\Omega_{z}(s)}=\frac{F_{LPFf}m_{y}\pi \lambda_{d}V_{com}}{V_{dac}K_{FBy}K_{DA}K_{PX}\alpha_{d}\left|K_{XVD}\right|}$$

So, the scale factor can be determined by *V_com_*.

### 3.3. Scale Factor Temperature Compensation

The scale factor temperature coefficient is tested on the experiment platform (which will be introduced in Chapter 4), and three repeatability tests are done (the results are shown in Table 2) and the temperature coefficient values of the three tests are 0.002631 mV/(°/s/°C) (variation 3.697%), 0.002641 mV/(°/s/°C) (variation 3.712%) and 0.002588 mV/(°/s/°C) (variation 3.633%), respectively. Then the average of the scale factor temperature coefficient is 0.00262 mV/(°/s/°C) (variation 3.680% with negative rate of change), which is the aim of the compensation. 

**Figure 6:**
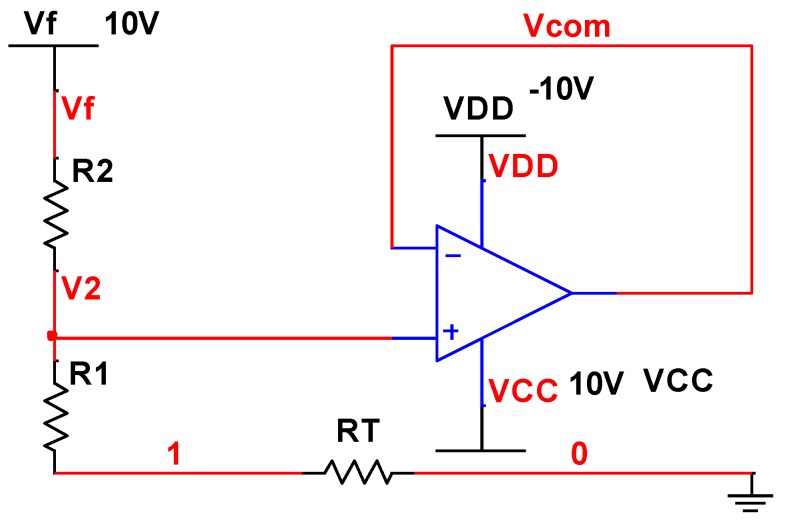
MEMS gyroscope scale factor temperature compensation circuit.

The MEMS gyroscope scale factor temperature compensation circuit is shown in Figure 6. The MEMS gyroscope scale factor temperature compensation circuit transform function can be expressed with the following equations:

(15) $$\left\{ \begin{array}{l} V_{2}=V_{com}\\ \frac{V_{f}-V_{2}}{R_{2}}=\frac{V_{2}}{R_{1}+R_{T}} \end{array}\right.$$

Then, Equation (16) can be obtained, and the parameters are shown in Table 3.

(16) $$\frac{V_{com}}{V_{f}}=\frac{R_{1}+R_{T}}{R_{1}+R_{T}+R_{2}}$$

**Figure 7:**
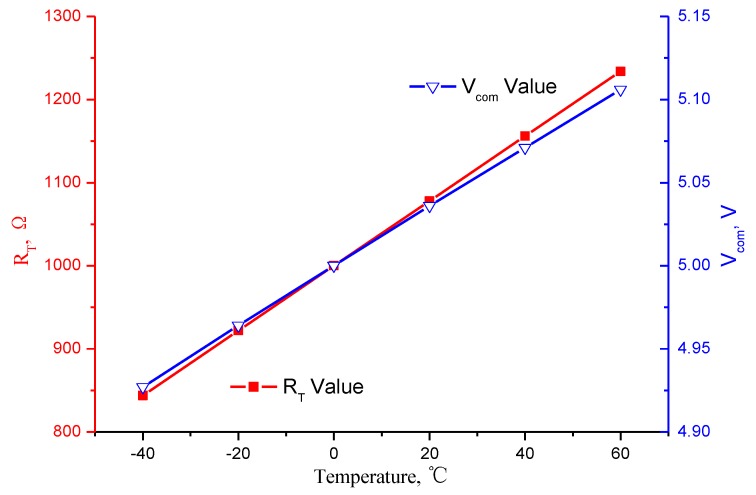
MEMS gyroscope scale factor temperature compensation circuit simulation result curves.

The simulation curve is shown in Figure 7, and the results show that the variation of *V_com_* within the temperature range from −40 °C to 60 °C is 3.633% and with a positive rate of change, which can compensate the negative rate of the gyroscope scale factor temperature coefficient. 

### 3.4. Temperature Bias Compensation

After the scale factor temperature compensation, the temperature bias experiments are conducted on the experimental platform, three repeatability tests are also done (the results are shown in Table 4), and the temperature bias coefficient values of the three tests are 0.0381 °/(s·°C) (variation 3.749%), 0.0407 °/(s·°C) (variation 4.004%) and 0.0396 °/(s·°C) (variation 3.887%), respectively. Then, the average of the temperature bias coefficient is 0.0395 °/(s·°C) (variation 3.880% with a negative rate of change), which is the aim of the compensation. 

**Figure 8:**
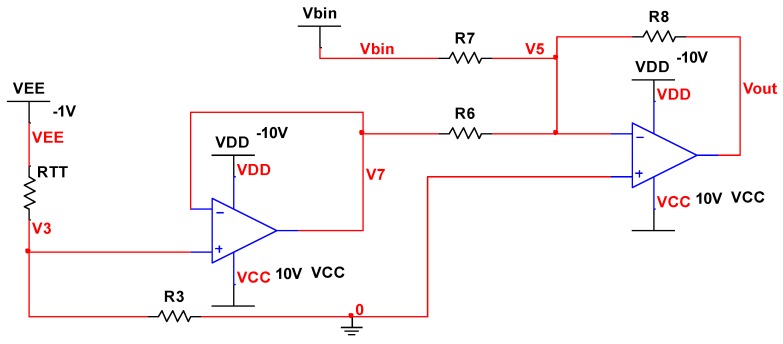
MEMS gyroscope temperature bias compensation circuit.

The temperature bias compensation circuit is shown in Figure 8; the parameters and simulation results are shown in Table 5, and the following functions can be obtained:

(17) $$\left\{ \begin{array}{l} \frac{V_{EE}-V_{3}}{R_{TT}}=\frac{V_{3}}{R_{3}}\\ V_{3}=V_{7}\\ V_{5}=0\\ \frac{V_{bin}-V_{5}}{R_{7}}+\frac{V_{7}-V_{5}}{R_{6}}=\frac{V_{5}-V_{out}}{R_{8}} \end{array}\right.$$

Then, the transfer function of the circuit can be expressed as

(18) $$V_{out}=-V_{bin}-\frac{V_{EE}R_{3}}{R_{3}+R_{TT}}$$

## 4. Temperature Compensation Experiments

**Figure 9:**
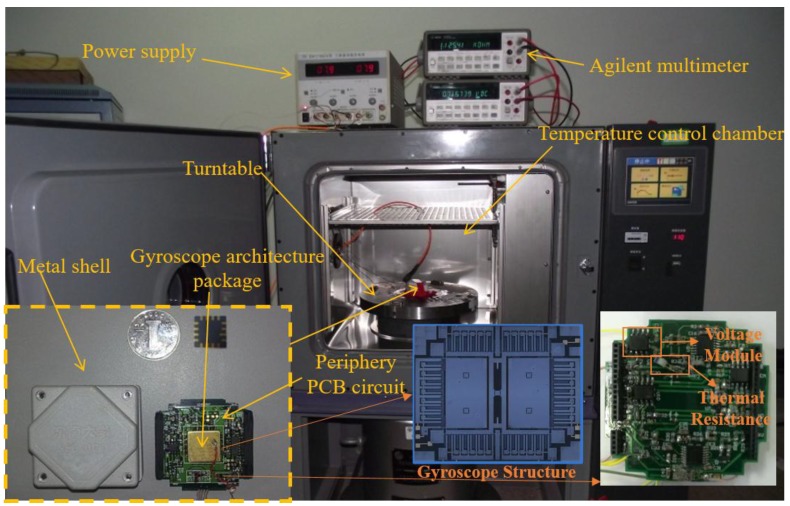
MEMS gyroscope porotype and temperature experiment platform.

The MEMS gyroscope is fixed on the turntable in a temperature chamber, which is shown in Figure 9. The turntable is employed to calibrate the scale factor of the gyroscope, and the temperature chamber is utilized to provide different temperature environments. The power supply is used to provide +10 V, −10 V and ground voltage, and the multimeter is used to pick up the output signal of the gyroscope. Temperature variable resistors *R_T_* and *R_TT_* are employed in the “Scale Factor” and “Bias Com” modules to reduce the scale factor and temperature bias drift. Another temperature-variable resistor, *R_tr_*, is employed to measure the real-time temperature inside the gyroscope shell, and the temperature value is picked up with the *V_out_* value synchronously. The temperature range in this paper is set as −40 °C to 60 °C, and the scale factor is tested every 20 °C. Each temperature experiment is repeated three times to verify the repeatability of the method.

The temperature compensation process is divided into three steps:

Firstly, the temperature drift of the scale factor is tested based on which voltage module parameters are set (which has already been discussed in Section 3.3).

**Figure 10:**
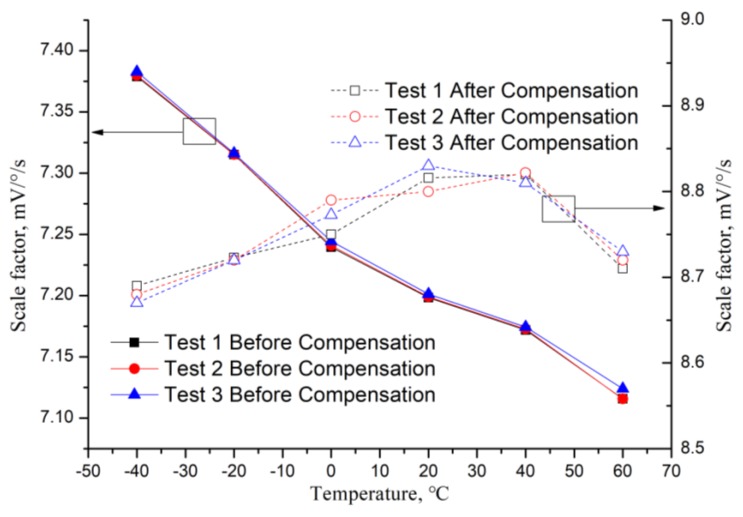
MEMS gyroscope scale factor before and after temperature compensation results.

Secondly, the scale factor temperature compensation method is tested, and the result curves are shown in Figure 10; also, three repeatability experiments are finished, and the variation of the three experiments are 1.485%, 1.623% and 1.824%, respectively, with an average value of 1.577%.

Thirdly, the temperature bias drift is tested based on which voltage module parameters are set (which has already been discussed in Section 3.4).

**Figure 11:**
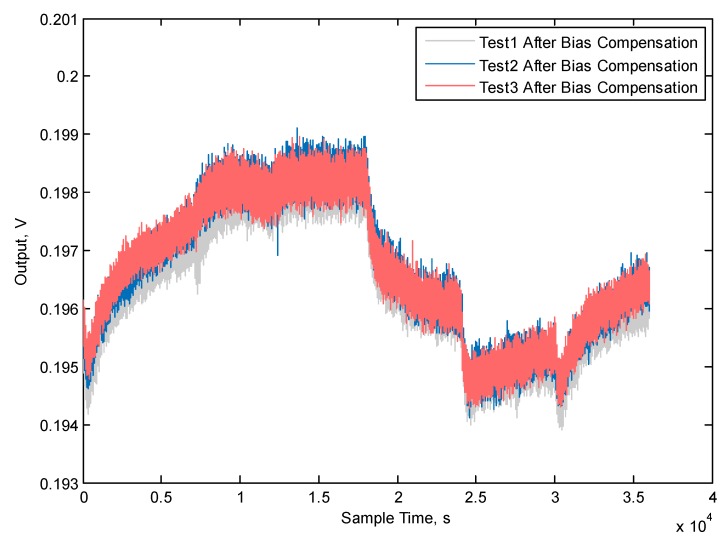
MEMS gyroscope temperature bias compensation results.

**Figure 12:**
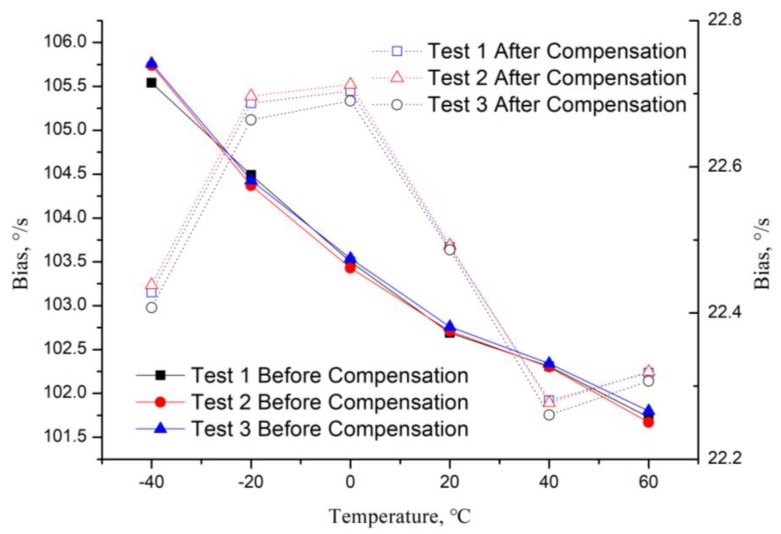
MEMS gyroscope bias before and after temperature compensation.

**Figure 13:**
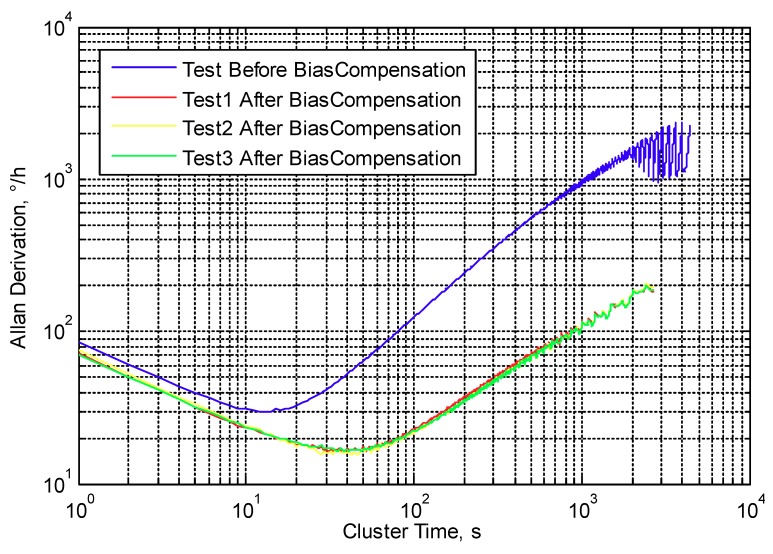
Allan derivation curves of MEMS gyroscope bias before and after temperature compensation.

Fourthly, the temperature bias compensation method is applied, and the three repeatability results are shown in Figure 11; the curves show that the repeatability of the compensation method is good. The results of bias before and after temperature compensation are shown in Figure 12. The variation of the three bias temperature compensation experiments are 1.914%, 1.868% and 1.912%, respectively, and the average value is 1.913%. The Allan derivation curves before and after temperature variation are shown in Figure 13, and the full-temperature bias stability (BS) and angular rate walking (ARW) parameters also improved from 29.52 °/h to 19.59 °/h and from 1.43 °/h/√Hz to 1.20 °/h/√Hz, respectively. Table 6 shows MEMS gyroscope temperature compensation test result.

## 5. Conclusions

In this study, the MEMS gyroscope temperature compensation method is investigated by using drive mode vibration characteristic compensation. The gyroscope working principle including drive mode and sense mode loops are analyzed, and the drive loop amplitude controlling voltage reference is set as the compensation point. Based on this, the scale factor temperature compensation circuit is designed and simulated. Then, the output level of the sense loop is investigated, and the temperature bias compensation circuit is designed and simulated. After that, temperature experiments are arranged, and the results show that, using the method proposed in this paper, the variation of the scale factor improves from 3.680% to 1.577% with a temperature range from −40 °C to 60 °C (enhanced by 57.14%). Furthermore, the bias variation improves from 3.880% to 1.930% (enhanced by 52.25%). The bias stability and angular rate walking parameter are also optimized (45.97% and 16.08%) in the benefit of the scale factor improvement. The experiment results verify the method proposed in this paper. 

## Figures and Tables

**Table 1:** MEMS gyroscope mechanical parameters under different temperatures (tested values).

Temperature (°C)	*ω_x_*_1_*/*2π (Hz)	*ω_x_*_2_*/*2π (Hz)	*ω_y_*_2_*/*2π (Hz)	*ω_y_*_1_*/*2π (Hz)	*Q_x_*_1_	*Q_x_*_2_	*Q_y_*_2_	*Q_y_*_1_
−40	2730.2	3494.7	3470.8	3367.2	2115	1773	1559	1415
−30	2729.8	3494.2	3470.1	3366.3	2044	1707	1495	1349
−20	2729.0	3493.1	3468.9	3365	1970	1626	1426	1272
−10	2728.1	3492.1	3467.9	3363.9	1900	1553	1362	1202
0	2727.2	3491.5	3467.0	3363.1	1850	1502	1315	1152
10	2726.8	3490.1	3465.5	3361.5	1804	1454	1275	1107
20	2726.2	3488.9	3464.1	3360.1	1744	1395	1224	1051
30	2725.4	3488.4	3463.6	3359.5	1713	1367	1199	1024
40	2724.1	3487.5	3462.6	3358.5	1658	1315	1151	973
50	2722.9	3486.2	3461.3	3357	1635	1290	1130	950
60	2721.3	3485.7	3460.7	3356.3	1625	1278	1122	939

**Table 2:** MEMS gyroscope scale factor temperature coefficient test results.

Temperature °C	Scale Factor Value mV/(°/s)			Temperature Coefficient mV/(°/s/°C)
	Test 1	Test 2	Test 3	
60	7.1158	7.1157	7.1239	$\begin{array}{l} Average\left[\frac{Max\;Value-Min\;Value}{60{}^{\circ}C-(-40{}^{\circ}C)}\right]\\ =0.00262\; \end{array}$*
40	7.1720	7.1728	7.1744	
20	7.1983	7.1990	7.2012	
0	7.2396	7.2409	7.2445	
−20	7.3151	7.3153	7.3163	
−40	7.3789	7.3798	7.3827	

* “*Value*” is the scale factor value, and “*Average*” is the average of tests 1, 2 and 3.

**Table 3:** MEMS gyroscope scale factor temperature compensation circuit parameters.

Parameter	Value	Parameter	Value
*R_T_* @60 °C	1234 Ω	*V_com_* @60 °C	5.106 V
*R_T_* @40 °C	1156 Ω	*V_com_* @40 °C	5.071 V
*R_T_* @20 °C	1078 Ω	*V_com_* @20 °C	5.036 V
*R_T_* @0 °C	1000 Ω	*V_com_* @0 °C	5.000 V
*R_T_* @−20 °C	922 Ω	*V_com_* @−20 °C	4.964 V
*R_T_* @−40 °C	844 Ω	*V_com_* @−40 °C	4.927 V
*V_f_*	10 V	*R*_1_	4400 Ω
*-*	-	*R*_2_	5400 Ω

**Table 4:** MEMS gyroscope temperature bias coefficient test result.

Temperature °C	Bias Value (*V_bin_*) °/s			Temperature Coefficient °/(s·°C)
	Test 1	Test 2	Test 3	
60	101.73	101.67	101.80	$\begin{array}{l} Average\left[\frac{Max\;Value-Min\;Value}{60{}^{\circ}C-(-40{}^{\circ}C)}\right]\\ =0.0395\; \end{array}$*
40	102.31	102.30	102.34	
20	102.69	102.71	102.76	
0	103.50	103.43	103.54	
−20	104.49	104.37	104.43	
−40	105.54	105.74	105.76	

* “*Value*” is the bias value *V_bin_*, and “*Average*” is the average of tests 1, 2 and 3.

**Table 5:** MEMS gyroscope temperature bias compensation circuit parameters.

Parameter	Value	Parameter	Value
*R_TT_* @60 °C	1234 Ω	*V_out_* @60 °C	194.739 mV
*R_TT_* @40 °C	1156 Ω	*V_out_* @40 °C	196.115 mV
*R_TT_* @20 °C	1078 Ω	*V_out_* @20 °C	198.555 mV
*R_TT_* @0 °C	1000 Ω	*V_out_* @0 °C	199.061 mV
*R_TT_* @−20 °C	922 Ω	*V_out_* @−20 °C	197.633 mV
*R_TT_* @−40 °C	844 Ω	*V_out_* @−40 °C	194.272 mV
*V_EE_*	−1 V	*R*_7_	10,000 Ω
*R*_3_	12,000 Ω	*R*_8_	10,000 Ω
*R*_6_	10,000 Ω	-	-

**Table 6:** MEMS gyroscope temperature compensation test result.

Temperature °C	Scale Factor Value mV/(°/s)					Bias Value °/s				
	Before Compensation	After Compensation				Before Compensation	After Compensation			
		Test 1	Test 2	Test 3	Average		Test 1	Test 2	Test 3	Average
60	7.1185	8.7110	8.7203	8.7300	8.7204	101.73	22.31	22.32	22.32	22.31
40	7.1731	8.8202	8.8220	8.8111	8.8178	102.32	22.26	22.28	22.28	22.27
20	7.1995	8.8160	8.8010	8.8302	8.8157	102.72	22.49	22.49	22.49	22.49
0	7.2417	8.7504	8.7903	8.7730	8.7712	103.49	22.69	22.70	22.71	22.70
−20	7.3156	8.7231	8.7208	8.7213	8.7217	104.43	22.66	22.69	22.70	22.68
−40	7.3805	8.6911	8.6811	8.6705	8.6809	105.68	22.41	22.43	22.44	22.42
**Variation**	**3.680%**	1.485%	1.623%	1.842%	**1.577%**	**3.880%**	1.914%	1.868%	1.912%	**1.913%**
**Bias Stability** (°/h)						**29.52**	16.05	15.59	16.21	**15.95**
**Angular Rate Walking** (°/h/√Hz)						**1.43**	1.19	1.24	1.18	**1.20**
